# Research IT maturity models for academic health centers: Early development and initial evaluation

**DOI:** 10.1017/cts.2018.339

**Published:** 2019-02-05

**Authors:** Boyd M. Knosp, William K. Barnett, Nicholas R. Anderson, Peter J. Embi

**Affiliations:** 1 Roy J. and Lucille A. Carver College of Medicine and the Institute for Clinical and Translational Science, University of Iowa, Iowa City, IA, USA; 2 Regenstrief Institute Inc., Indiana, CTSI, Indiana University School of Medicine, Indianapolis, IN, USA; 3 Clinical Translational Science Center and Department of Public Health Sciences, UC Davis Health System, University of California, Davis, Davis, CA, USA

**Keywords:** Maturity models, research informatics, research information technology, biomedical informatics, CTSA.

## Abstract

This paper proposes the creation and application of maturity models to guide institutional strategic investment in research informatics and information technology (research IT) and to provide the ability to measure readiness for clinical and research infrastructure as well as sustainability of expertise. Conducting effective and efficient research in health science increasingly relies upon robust research IT systems and capabilities. Academic health centers are increasing investments in health IT systems to address operational pressures, including rapidly growing data, technological advances, and increasing security and regulatory challenges associated with data access requirements. Current approaches for planning and investment in research IT infrastructure vary across institutions and lack comparable guidance for evaluating investments, resulting in inconsistent approaches to research IT implementation across peer academic health centers as well as uncertainty in linking research IT investments to institutional goals. Maturity models address these issues through coupling the assessment of current organizational state with readiness for deployment of potential research IT investment, which can inform leadership strategy. Pilot work in maturity model development has ranged from using them as a catalyst for engaging medical school IT leaders in planning at a single institution to developing initial maturity indices that have been applied and refined across peer medical schools.

## Introduction

Conducting and advancing biomedical research has long been and remains an essential part of the mission of academic health centers (AHCs). Recent and ongoing initiatives focused on accelerating clinical, translational, and foundational biomedical research have led AHCs to invest in infrastructure and capabilities that enable and support research activities which are increasingly information and data management-intensive [1–3]. Research informatics and information technology (research IT) encompasses technological, human and organizational resources, systems and methods that manage and analyze data, information and knowledge to improve biomedical and health research [4]. The success and growth of institutions’ research enterprise relies upon advanced research IT solutions and capabilities, with investments in such infrastructure steadily rising [5, 6]. Indeed, research IT services are now at the heart of AHC research capabilities, and are integral to supporting broad and expanding research initiatives in population health, genomics, imaging, personalized medicine, as well as increasingly integrated into comprehensive data security and data sharing strategies. These investments in technology and expertise remain unevenly aligned with clinical mission investments. To be competitive and successful in the current research environment, AHCs require thoughtful research IT investment strategies with measurable outcomes that can align with institutional clinical and operational goals. Increasingly, AHC institutional leaders are feeling pressured to strategically increase investment in research IT due to a number of issues, including, but not limited to:

- Institutional competitiveness for national initiatives such as precision health, patient-centered outcomes research that require significant, sustainable investments in research IT skills and resources [7–10].
- Health IT systems have a growing role in not only enabling care and operations, but also enabling research and learning health systems, and this can raise new risk and compliance considerations related to security and data sharing strategies.
- Programs driving clinical and translational science require more efficient and effective models of access to data and IT systems.
- Research funders increasingly expect institutional support for research IT infrastructure and services as prerequisites to funding.

As the demand for such infrastructure and services increases, challenges in how to maintain effective oversight and support for research IT expand at a similar pace, with less clear measures of success. The distributed and variable nature of research funding and productivity often requires investment and deployment without the benefit of predictable funding streams that are core to the strategic development of the clinical and educational missions of AHCs. Without a similar predictable funding stream, many organizations find themselves experimenting with different strategies for implementing research IT services, which makes recruiting and retaining the skilled research IT professionals (e.g., data scientists, research informaticians, and support staff) challenging, given the limited workforce and the growing demand [11]. Other challenges relate to emerging but not-yet-widespread models for research IT governance at AHCs, ongoing requirements related to leveraging health IT systems and data for secondary research purposes, and mechanisms for incentivizing participation in evidence-generating activities for personnel and organizations not part of the traditional research enterprise [12–16]. As institutions address requirements for increasing their research IT investments, there is a need to better characterize their shared value and contributions to the health IT stakeholders. Institutions need guidance on how best to leverage these investments within institutional priorities and strategies, promote their strengths in the context of the national landscape of research IT maturity, and strategically manage external forces such as regulatory requirements, data sharing requirements, and emerging initiatives of sponsors.

## Approach

Recent studies show that research IT capabilities and resources are inconsistently implemented across AHCs. This often relates to the differences in overall organization of AHCs [17–19], such as the difference between a single organization governing all three missions versus one where governance is separated. It can also be seen in the inconsistent presence of a formal aligned department of biomedical informatics or the chief research information officer role [20].

Maturity models [21] have been shown to be effective tools in guiding organizational development for prioritization and process improvement in a wide range of areas of information technology [22]. Maturity models assume that organizations, processes, and services develop through a series of stages of increasing quality and predictability [23, 24]. Each stage is modeled to be more efficient and effective than the previous stage. A key tenet of maturity models is that an organization needs to pass through each stage to reach a higher next stage. The path provided by these incrementally improving stages creates achievable steps for improvement as the organization progresses from one stage to the next working towards an ideal future state for the organization, which may or may not be the highest level of maturity due to considerations of costs, culture, and the impact on other missions.

Maturity models are an adaptable framework and may be applied to a single team, across the entire organization or across multiple organizations. They may be used to assess an organization’s capacity to deliver a service—providing insight into organizational culture, policy, and structure—or to measure the degree to which an institution has deployed technologies relative to delivering a service. These two characteristics of maturity models—the ability to enable incremental improvements towards an ideal state and the adaptability of the frameworks—make them a promising tool for helping grapple with the research IT complexities and inconsistent implementations seen in academic medicine.

Maturity models have provided a framework for building indices to look at electronic health record (EHR) deployment [25]. The Health Information Management Systems Society tools have been effective in guiding the implementation of expensive and complex EHRs by establishing a standard for assessing deployment of the EHRs—by measuring levels of service implementation. Maturity has also been applied to various areas of IT service development in higher education [26]. Educause has developed both a deployment index for various areas of IT but has also created indices that look at organizational capacity for delivering services. These two applications—EHRs and higher Ed IT—inspire the idea of applying maturity to research IT in academic medicine.

### Establishing Institutional Metrics and Standards for Research IT

We propose the creation and application of maturity models to guide institutional investment in research IT. There appears to be ample information in the AHC IT and informatics community to build indices that would allow organizations to do their own self-assessments, while also working towards establishing standards from interinstitutional data.

### The Need for Two Types of Indices

We suggest a need for two types of tools, a maturity index and a deployment index [26]. A maturity index measures organizational capacity to deliver a service, considering multiple factors including culture, policy, and organization. A deployment index measures the degree to which an institution has implemented a technology related to delivering a service.

Maturity indices address broader often nontechnical organizational issues and assessments can be completed by multiple offices (e.g., finance, scientific affairs, IT) in an organization to determine variance of perception within an organization and to build consensus on what an organization’s “ideal state” for research IT is. Maturity indices also serve as effective comparators with peer institutions.

**Fig. 1:**
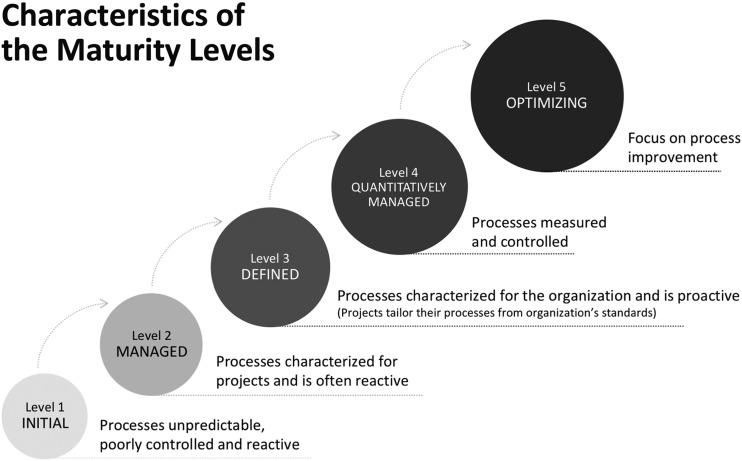
Stages (levels) of maturity.

Deployment indices allow the assessment of a given technology (e.g., research storage, clinical trials management system) against a classic maturity scale (Fig. 1). In an initial version of a deployment index, people doing the assessment could simply rate their deployment against a maturity scale. In a future version of the index assessment, there could be questions developed to determine quality, comprehensiveness, and complexity levels of a service. As with the maturity index, deployment index results could be effective tools for both internal and external planning. Deployment indices require a higher level of technical familiarity and understanding than maturity indices complete effectively.

## Development and Initial Experiences

The authors have been involved in developing and testing maturity models for research IT in a number of venues. Embi has led discussions of maturity models at the Clinical Research Forum IT Roundtable where he presented a basic framework for a research IT maturity model. Embi’s framework has been built on by the other authors. One (Anderson) used a variant of this model to engage IT and medical center leadership in a planning exercise about research IT at his institution, building consensus on what research capabilities exist and establishing a common framework for planning. In this work, Anderson tested a maturity index at the University of California Davis to assess the capabilities for development of strategic research IT. To identify both infrastructure capabilities as well as current leverage of these resources for scientific applications, two assessments independently rated research IT and translational informatics capabilities from 22 stakeholders including chief information officer (CIO), associate dean and director-level roles. The study initially sought to test both maturity and deployment indices, but found that the challenges in developing common frameworks across such a broad campus required focusing on the maturity index as an initial framework to socialize the class of resources and stakeholders. UC Davis is intending to extend this work to a regular assessment.

Barnett and Knosp engaged the Association of American Medical Colleges Group on Information Resources to build two indices—a maturity index and a deployment index (online Supplementary Appendix 1 and 2) using methodologies developed by Educause researchers [26]. They held focus groups and engaged subject matter experts to build a maturity index and a deployment index for research IT at AHCs. A pilot study was performed with members of the Group on Information Resources. The maturity index was used to stimulate a discussion at the 2017 Association of American Medical Colleges Group on Business Affairs Principal Business Officers that includes responses from AHC financial officers, senior research officers and CIOs. Both indices were presented and reviewed at the 2017 Clinical Research Forum IT Roundtable in Chicago.

**Fig. 2:**
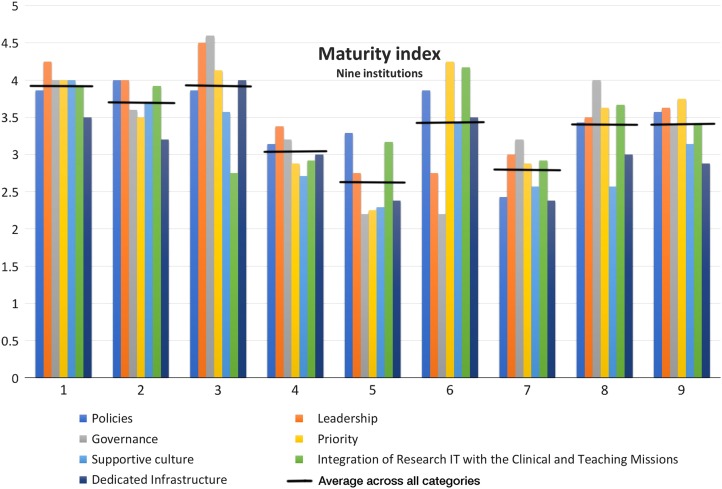
Example of maturity index results for seven categories of maturity. Horizontal line indicates average across all categories. IT, information technology.

**Fig. 3:**
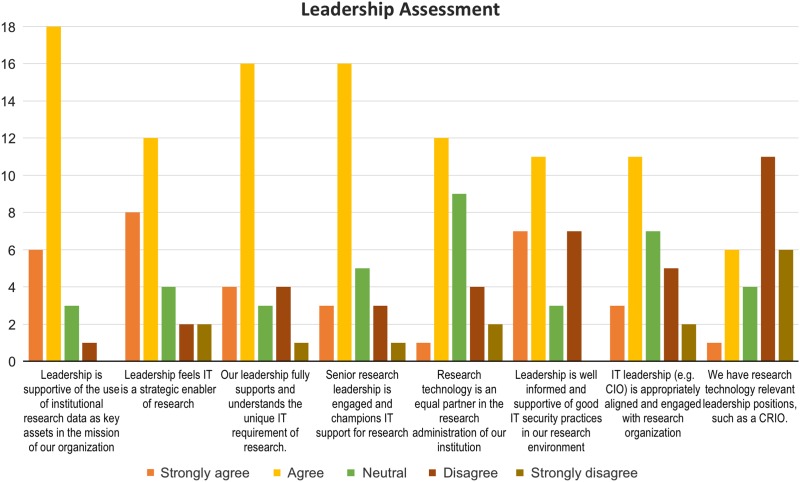
Example of maturity index results for leadership category showing counts of Likert response for each question in the category. CIO, chief information officer; CRIO, chief research information officer; IT, information technology.

**Fig. 4:**
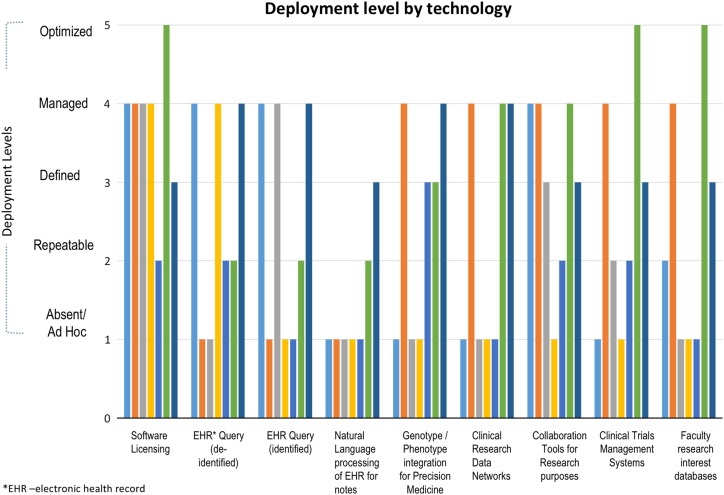
Deployment index results. Each color is a different institution.

The maturity index developed was an assessment of organizational characteristics using a Likert scale in seven categories including policies, governance, priority, leadership, supportive culture, integration with other missions and dedication infrastructure (Fig. 2). Fig. 2 shows the category and overall index scores across nine institutions and Fig. 3 shows the counts for Likert responses for one category—leadership. Looking at the data in these two different ways—single scores or raw responses provides insights at different levels of details into how research IT is viewed at these different organizations. The deployment index was a list of technologies with users selecting the maturity level the technology has been deployed at their institutions (Fig. 4). Looking at this data provides a quick assessment of institutional areas of strength or in need of development and could inform communities about topics of interest (e.g., common areas that need development).

### Value Proposition and Impact

Ultimately, once fully refined, validated and widely implemented, the value proposition of research IT maturity and deployment indices will be demonstrated by their use enabling AHCs to define strategic investment into and development of research IT services and capabilities. Indeed, when put into widespread use, such maturity and deployment models would serve the greater biomedical research community in a variety of ways. Some potential examples and impacts include:

- Single enterprise identification of gaps and readiness in aligning clinical needs with research.
- Enabling and optimizing research environments that cross organizational boundaries (e.g., computational biology and clinical imaging, patient-centered mobile health, and practice networks).
- Providing comparable metrics for funding organizations to allow for review of institutional research IT readiness against aggregated community measures.
- Developing guidelines for specific readiness and deployment indices as part of emerging communities of practice (e.g., multi-site clinical/research networks, precision health, or artificial intelligence in medicine initiatives).
- Establishing effective representation of local expertise and infrastructure capabilities for strategic review, academic recruitment, or development of public-private partnerships.

## Conclusions

Based on this pilot work, maturity models applied to research IT have shown the potential to inform a range of leadership stakeholders within institutions in a number of ways, including creating a guide for implementation and evaluation, providing a platform and context for internal discussion and planning, and defining organizational best practices for research IT support within academic medicine.

Next steps include establishing common open source indices for institutions to use across the value categories described above and to create objective maturity and deployment indices for specific services aligned with strategic needs.

Across these activities, it will be critical to continuously improve indices through building on the feedback from institutions and groups that use them and through establishing means to share and validate data and outcomes. As we move beyond pilot initiatives to broader data collection and use, we hope to be able to correlate output of these indices with other markers of maturity such as research productivity and emerging metrics for research IT.

## Funding/Support

This project was supported by the National Institutes of Health, National Center for Advancing Translational Sciences, Clinical and Translational Sciences Awards including grant numbers UL1TR001860, U54TR001356, and UL1TR001108.

## Disclosures

The authors have no conflicts of interest to declare.

## ORCID ID

Boyd Knosp https://orcid.org/0000-0002-3834-3135.

## Supplementary material

For supplementary material accompanying this paper visit https://doi.org/10.1017/cts.2018.339. click here to view supplementary material

## References

[1] Stephens K, et al Implementing partnership-driven clinical federated electronic health record data sharing networks. International Journal of Medical Informatics 2016; 93: 26–33. 27435944 10.1016/j.ijmedinf.2016.05.008 PMC6790180

[2] Embi PJ, Payne PR. Evidence generating medicine: redefining the research-practice relationship to complete the evidence cycle. Medical Care 2013; 51(Suppl. 3): S87–S91. 23793052 10.1097/MLR.0b013e31829b1d66

[3] Smoyer WE, Embi PJ, Moffatt-Bruce S. Creating local learning health systems: think globally, act locally. JAMA 2016; 316: 2481–2482. 27997662 10.1001/jama.2016.16459

[4] Bernstam EV, et al Synergies and distinctions between computational disciplines in biomedical research: perspective from the clinical and translational science award programs. Academic Medicine 2009; 84: 964–970. 19550198 10.1097/ACM.0b013e3181a8144d PMC2884382

[5] Embi PJ, Payne PR. Clinical research informatics: challenges, opportunities and definition for an emerging domain. Journal of the American Medical Informatics Association 2009; 16: 316–327. 19261934 10.1197/jamia.M3005 PMC2732242

[6] Murphy SN, et al Current state of information technologies for the clinical research enterprise across academic medical centers. Clinical and Translational Science 2012; 5: 281–284. 22686207 10.1111/j.1752-8062.2011.00387.x PMC5439868

[7] **National Cancer Institute** Cancer Moonshot Blue Ribbon Panel [Internet], 2018 [cited Oct 14, 2018]. (https://www.cancer.gov/research/key-initiatives/moonshot-cancer-initiative/blue-ribbon-panel)

[8] **Clinical & Translational Science Awards Program, Center for Leading Innovation & Collaboration** Established common metrics [Internet], 2018 [cited Oct 14, 2018]. (https://clic-ctsa.org/common_metrics/established-common-metrics)

[9] **National Institutes of Health** NIH data sharing policy [Internet], 2018 [cited Oct 14, 2018]. (https://grants.nih.gov/grants/policy/data_sharing/)

[10] **National Institutes of Health** NIH strategic plan for data science [Internet], 2018 [cited Oct 14, 2018]. (https://datascience.nih.gov/sites/default/files/NIH_Strategic_Plan_for_Data_Science_Final_508.pdf)

[11] Bowley R. Linked in official blog, the fastest-growing jobs in the U.S. based on linkedin data [Internet], 2017 [cited Oct 14, 2018]. (https://blog.linkedin.com/2017/december/7/the-fastest-growing-jobs-in-the-u-s-based-on-linkedin-data)

[12] Embi PJ, et al Integrating governance of research informatics and health care IT across an enterprise: experiences from the trenches. AMIA Joint Summits on Translational Science Proceedings 2013; 2013: 60–62. PMC3845750 24303236

[13] Hersh WR, et al Caveats for the use of operational electronic health record data in comparative effectiveness research. Medical Care 2013; 51(Suppl. 3): S30–S37. 23774517 10.1097/MLR.0b013e31829b1dbd PMC3748381

[14] Embi PJ, Tsevat J. Commentary: the relative research unit: providing incentives for clinician participation in research activities. Academic Medicine 2012; 87: 11–14. 22201633 10.1097/ACM.0b013e31823a8d99 PMC3914136

[15] Payne PR, Embi PJ, Sen CK. Translational informatics: enabling high-throughput research paradigms. Physiological Genomics 2009; 39: 131–140. 19737991 10.1152/physiolgenomics.00050.2009 PMC2789669

[16] Weiner MG, Embi PJ. Toward reuse of clinical data for research and quality improvement: the end of the beginning? Annals of Internal Medicine 2009; 151: 359–360. 19638404 10.7326/0003-4819-151-5-200909010-00141

[17] Obeid J, et al Sustainability considerations for clinical and translational research informatics infrastructure. Journal of Clinical and Translational Science, doi:10.1017/cts.2018.332. 10.1017/cts.2018.332 PMC6390401 30828467

[18] Wilcox A, et al Sustainability considerations for health research and analytic data infrastructures. eGEMs 2014; 2: 1113. 25848610 10.13063/2327-9214.1113 PMC4371522

[19] Payne PR, et al People, organizational, and leadership factors impacting informatics support for clinical and translational research. BMC Medical Informatics and Decision Making 2013; 13: 20. 23388243 10.1186/1472-6947-13-20 PMC3577661

[20] Sanchez-Pinto LN, et al The emerging role of the chief research informatics officer in academic health centers. Applied Clinical Informatics 2017; 8: 845–853. 28832068 10.4338/ACI-2017-04-RA-0062 PMC6220697

[21] Nolan R. Managing the computer resource: a stage hypothesis. Communications of the ACM 1973; 16: 399–405.

[22] Mutafelija B, Stromberg H. Systematic Process Improvement Using ISO 9001:2000 and CMMI. Boston, MA: Artech House, 2003.

[23] Carvalho JV, Rocha A, Abreu A. Maturity models of healthcare information systems and technologies: a literature review. Journal of Medical Systems 2016; 40: 131. 27083575 10.1007/s10916-016-0486-5

[24] Crosby P. Quality is Free. New York: McGraw-Hill, 1979.

[25] **HIMSS** HIMSS analytics [Internet], 2017 [cited Oct 14, 2018]. (http://www.himssanalytics.org/)

[26] Grajek S. The digitization of higher education: charting the course. *EDUCAUSE Review* [Internet], 2016 [cited Oct 14, 2018]. (https://er.educause.edu/articles/2016/12/the-digitization-of-higher-education-charting-the-course)

