# Supplementary material for: Research IT maturity models for academic health centers: Early development and initial evaluation
Source: J Clin Transl Sci. 2019 Feb 5;2(5):289–94. doi: 10.1017/cts.2018.339 (PMC6390403; doi:10.1017/cts.2018.339)
Supplement: Supplementary file 1 [file S2059866118003394sup.zip › S2059866118003394sup001.pdf]

Research Technology Maturity Index

Pilot Maturity index for Research technology in Academic Medical Centers
A maturity index (MI): measures organizational capacity to deliver a service, taking into account multiple factors, including culture, policy, and organization.

This is a pilot index and its primary purpose is to help develop an instrument to be made available to institutions involved in biomedical research. Your name and institution requested below is to have a record of who has responded to this survey and will not be published or shared in any way.

First name ______________________________

Last name ______________________________

E-mail ______________________________

Institution Name ______________________________

****

Select best response For each statement pick the best response regarding your institution, from your perspective.

Policies
These statements explore formal rules pertaining to research IT at your institution.

	Strongly Disagree	Disagree	Neutral	Agree	Strongly Agree
Our academic medical center has established polices to govern research-specific data management.	○	○	○	○	○
Our institution technology policies address resarch use.	○	○	○	○	○
Our policies regarding research technology and research data management are reviewed on a regular basis.	○	○	○	○	○
Our institution has a framework for establishing agreements for data use and sharing.	○	○	○	○	○
Our institution practices and/or policies align with NIH policies for data sharing.	○	○	○	○	○
We have effective enforcement consequences for violators of research data policies.	○	○	○	○	○
We have a unit that is responsible for operationalizing our policies for research IT.	○	○	○	○	○

Leadership
This set of statements explores leadership awareness and engagement in research IT.

	Strongly Disagree	Disagree	Neutral	Agree	Strongly Agree
Leadership is supportive of the use of institutional research data as key assets in the mission of our organization	◯	◯	◯	◯	◯
Leadership feels IT is a strategic enabler of research	◯	◯	◯	◯	◯
Our leadership fully supports and understands the unique IT requirement of research.	◯	◯	◯	◯	◯
Senior research leadership is engaged and champions IT support for research	◯	◯	◯	◯	◯
Research technology is an equal partner in the research administration of our institution	◯	◯	◯	◯	◯
Leadership is well informed and supportive of good IT security practices in our research environment	◯	◯	◯	◯	◯
IT leadership (e.g. CIO) is appropriately aligned and engaged with research organization	◯	◯	◯	◯	◯
We have research technology relevant leadership positions, such as a CRIO.	◯	◯	◯	◯	◯

Governance
The set of statements explores organizational activites to make decisions about Research IT.

	Strongly Disagree	Disagree	Neutral	Agree	Strongly Agree
We have formal research IT governance structure that aligns with the overall organizational governance structure.	○	○	○	○	○
We have a faculty advisory group that provides input/guidance on research technology efforts.	○	○	○	○	○
Faculty play a role in decisions made about research IT strategic planning and research data	○	○	○	○	○
Senior leadership owns and engages in the governance regarding research IT and associate policies	○	○	○	○	○
Our organization has an effective way to make decisions about research IT issues and policies	○	○	○	○	○

Priority
This set of statements explores tangible ways that research IT is a priority for your institution.

	Strongly Disagree	Disagree	Neutral	Agree	Strongly Agree
Research has the same institutional priority as the clinical and educational missions	○	○	○	○	○
We have a roadmap for research technology	○	○	○	○	○
Support for research technology is part of our organization's strategic plan.	○	○	○	○	○
We have a dedicated Research IT support staff.	○	○	○	○	○
Research IT support is part of our enterprise IT budget.	○	○	○	○	○
Research IT support is part of our enterprise IT services.	○	○	○	○	○
We have funding for exploring innovative research technologies	○	○	○	○	○
Resources to support and develop research IT are supported in a sustainable way.	○	○	○	○	○

Supportive Culture
These statements explore opinions and processes at your organization impacting research IT.

	Strongly Disagree	Disagree	Neutral	Agree	Strongly Agree
Researchers know where to go for support for research IT issues.	○	○	○	○	○
Researchers at our institution are well informed regarding IT security issues and policies	○	○	○	○	○
IT support at our institution understands and addresses the special needs of our researchers	○	○	○	○	○
IT is structured to respond agilely to researcher requests	○	○	○	○	○
We have IT staff with domain expertise who are positioned to leverage this for supporting research.	○	○	○	○	○
We have ways to review proposed exemptions to security policies/practices in our research environment	○	○	○	○	○
Our organization is tolerant of investing in high-risk technologies to support research.	○	○	○	○	○

Integration of Research IT with the Clinical and Teaching missions
These statements explore points of intersection between research IT and the educational and clinical missions at your organization.

	Strongly Disagree	Disagree	Neutral	Agree	Strongly Agree
Our curriculum provides exposure to research IT and informatics methods.	○	○	○	○	○
Our education programs produce workers to fill research IT or informatics support positions.	○	○	○	○	○
Our educational researchers use current-state IT and informatics tools to manage and analyze educational data.	○	○	○	○	○
Our organization facilitates the capture of clinical data in the EMR for both healthcare delivery and research.	○	○	○	○	○
We have an insitutionally recognized and effective workflow for managing patient consent.	○	○	○	○	○
We make effective use of our EMR to enable recruitment of subjects	○	○	○	○	○
Clinical IT teams are well connected with research it teams	○	○	○	○	○
Our clinical enterprise IT supports the recruitment of subjects for clinical research and clinical trials	○	○	○	○	○
We have effective methods of tracking research relevant certification and skills of of our investigators and care coordinators	○	○	○	○	○
We have effective methods of tracking research-related care tasks within our clinical care workflows.	○	○	○	○	○

Our organization is committed to becoming a learning health organization	○	○	○	○	○
Our organization strives to enable the virtuous cycle of evidence generation through research Our organization strives to enable the virtuous cycle of evidence generation through research.	○	○	○	○	○

Dedicated infrastructure
These statements explore the availability and capacity for your institution's technology resources in support of research.

	Strongly Disagree	Disagree	Neutral	Agree	Strongly Agree
Researchers have efficient and accessible ways to query de-identified patient records for research	○	○	○	○	○
We have central data storage resources that allow individual researchers to comply with institutional and NIH data policies.	○	○	○	○	○
On-boarding processes allow timely access to campus resources for researchers new to our institution	○	○	○	○	○
We are well connected and contribute to multi-institutional networks to recruit subjects from a geographically disperse populations	○	○	○	○	○
We have a robust research technology service catalog and portfolio	○	○	○	○	○
We have adequate computational resources for our research needs.	○	○	○	○	○
We have adequate staff resources to provide IT support for specialized research instrumentation.	○	○	○	○	○
We have adequate analytics resources for our researchers.	○	○	○	○	○

***********Scores (1-5) Note : higher is more mature

Policies ______________________

Leadership ______________________

Governance ______________________

Priority ______________________

Supportive culture ______________________

Integration of Reesearch IT with the Clinical and Teaching Missions ______________________

Dedicated Infrastructure ______________________

Note: To keep an immediate copy of your responses and scores, you should print this page before hitting submit.

Please estimate how much time you spent filling out this index.

◯ less than 5 minutes
◯ 5-10 minutes
◯ 10-15 minutes
◯ over 15 minutes

Please provide any feedback regarding this index:

____
