# Supplementary material for: Research IT maturity models for academic health centers: Early development and initial evaluation
Source: J Clin Transl Sci. 2019 Feb 5;2(5):289–94. doi: 10.1017/cts.2018.339 (PMC6390403; doi:10.1017/cts.2018.339)
Supplement: Supplementary file 1 [file S2059866118003394sup.zip › S2059866118003394sup002.pdf]

Research IT Deployment Index

Pilot Deployment index for Research technology in Medical School Education A Deployment Index (DI): Measures the degree to which institution has deployed technologies related to delivering a service.Note: Name, email and institution are collected to track who has responded. This information will not be shared in any way.

Frst name __________________________________

Last name __________________________________

E-mail __________________________________

institution __________________________________

Rate technologies For this index, please rate the level you feel your institution has deployed the following technologies:

Research technologies This section has you rate the level of maturity that the following technologies that directly support research are implemented at your institution.

	Absent/Ad hoc	Repeatable	Defined	Managed	Optimized
1. Mass Storage	○	○	○	○	○
2. High Performance Computation	○	○	○	○	○
3.Support for research instrumentation in basic science labs	○	○	○	○	○
4. Data Capture Services	○	○	○	○	○
5. Bioinformatics tools (to process sequence and genetic data)	○	○	○	○	○
6. Biosample Tracking Databases	○	○	○	○	○
7. Research Imaging Databases	○	○	○	○	○
8. Genomics databases	○	○	○	○	○
9. Protocol Design	○	○	○	○	○
10. Registry Systems	○	○	○	○	○
11. Clinical Research Data Warehouse	○	○	○	○	○
12. Software Licensing Programs	○	○	○	○	○
13. Research and EMR integration (for patient safety, recruitment...)	○	○	○	○	○
14. EMR query tools for de-identified data	○	○	○	○	○
15. EMR Query tools for identified data	○	○	○	○	○
16. Natural Language Processing for EMR Notes	○	○	○	○	○

17. Systems for Genotype/Phenotype Integration for Precision Medicine	○	○	○	○	○
18. Clinical Research Data Networks	○	○	○	○	○
19. Collaboration Tools for Research Purposes	○	○	○	○	○
20. Telepresence	○	○	○	○	○

****

Administration Research Technologies This section has you rate the level of maturity that the following technologies that help plan, manage and track research at your institution.

	Absent/Ad hoc	Repeatable	Defined	Managed	Optimized
21. Clinical Trials management systems	○	○	○	○	○
22. Faculty research interest database	○	○	○	○	○
23. Grant submission tools	○	○	○	○	○
24. Internal pilot grant review tools	○	○	○	○	○
25. Research Service Management Tools	○	○	○	○	○
26. Electronic IRB submission and review tools	○	○	○	○	○

Please estimate how much time you spent filling out this index.

◯ less than 5 minutes
◯ 5-10 minutes
◯ 10-15 minutes
◯ over 15 minutes

Please provide any feedback regarding this index:

____
